# The use of evaluation methods for the overall assessment of health policy: potential and limitations

**DOI:** 10.1186/s12962-020-00238-4

**Published:** 2020-10-13

**Authors:** Krzysztof Kaczmarek, Piotr Romaniuk

**Affiliations:** Department of Health Policy, School of Health Sciences in Bytom, Medical University of Silesia in Katowice, ul. Piekarska 18, 41-902 Bytom, Poland

**Keywords:** Evaluation, Health policy, Public health

## Abstract

**Background:**

The implementation of public policies requires special attention from public authorities to ensure their transparency, effectiveness and efficiency. For this reason, efforts to evaluate the abovementioned politics gained attention and importance. Similar processes, by their very nature, are also noticeable in the area of health policy, however, the nature of the solutions used raises questions about the extent to which they allow to capture the assessed phenomenon in a holistic way. The current approach to the problem of evaluating health policy shows a tendency to break down this phenomenon into components including policies, programs or projects. The purpose of this publication was to assess the main methodological approaches used in evaluation studies in terms of their usefulness and limitations in conducting overall assessment of health policy.

**Main body:**

The publication divides evaluation methods based on three main criteria identified in the literature—time, purpose and scope of evaluation. Methodological approaches to evaluation activities detailed on this basis are discussed from the point of view of their characteristics, usefulness and limitations in the creation of comprehensive health policy assessments. The growing awareness of the need for a different approach to evaluation, which was confirmed by the course of the discourse on evaluation in public health, was also pointed out.

**Conclusion:**

Given the complexity of the matter making up the health policy, attempts aimed at its overall assessment should be based on an approach integrating many approaches, while ensuring coordination of evaluation activities that should be subject to common assumptions.

## Background

**Fig. 1:**
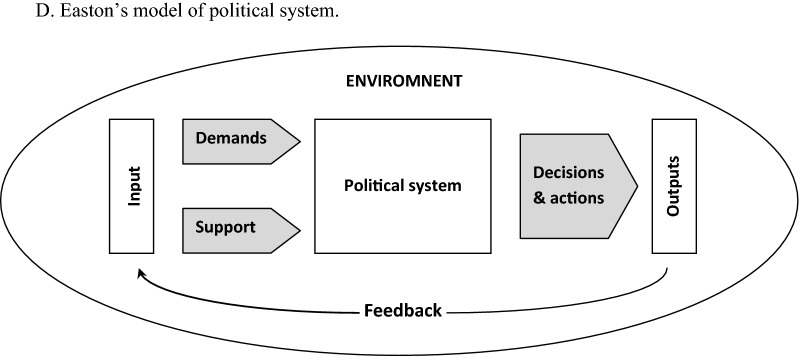
D. Easton’s model of political system

The process of implementing public policy is becoming an extremely demanding task at present day. This is not only due to the complexity of the matter in which the activities are carried out, nor to the degree of difficulty in achieving the objectives, although in both cases underestimating these factors would be an obvious mistake. Undoubtedly, the most important change that has taken place in recent decades is the change in the environment in which decision-making processes are undertaken. The increasingly widespread crisis of trust in representatives of public authorities, largely stimulated by greater possibilities of social control of decision-makers, stimulated on one hand a change in the way decisions are communicated, with emphasis being put on transparency of activities and a stronger focus on demonstrating their purpose. On the other hand, a layer of broadly understood economics of activities has acquired a very important meaning, which in turn is related to the growing concerns about the institutional, financial or normative capacity to meet the demand for healthcare and public health services. This phenomenon was aptly captured by David Easton, depicting the political system in the form of a whole, whose level of balance depends on the relationship between the inputs (impulses) which can be viewed as expression of social demands and expectations and the outputs (results) generated by the system in response to those demands (Fig. 1) [1]. As noted by Reichard, a characteristic feature of societies based on the principles of liberal democracy is pluralism, and thus a large variety of system entries (or inputs), while the system has limited capacity to respond to all incoming impulses, which leads to the formation of imbalances [2]. In the reality of scarcity of resources and inability to respond to all needs, effectiveness and rationality come to the fore. Hence, it can be stated without doubt that the expediency of spending on specific public policies will be subject to critical assessment from public opinion representatives. At the same time, the trend clearly leaving its mark on the contemporary political scene is the strong polarization of society manifested in the last few years. As a result, growing number of ideologically marked subjects is being introduced to the public debate, which in turn makes the axiological layer of implemented policies one of the main criteria for their assessment.

Health policy in its entirety is not an exception in this respect and falls subject to the abovementioned regularities, although it also has its own conditions that modify the severity of the outlined processes. Undoubtedly, if viewed from the perspective of developed countries, a factor of strong importance is the pressure from the growing demand for health services, which collides with the amount of available resources. The need to demonstrate the legitimacy of spending on specific activities (policies) implemented in the area of health, means that more attention of both decision-makers and researchers has been directed towards evaluation as an area of activities that can potentially be a solution to the discussed problems.

Conducting a discourse dedicated to evaluation in the context of health policy requires the specification of several concepts, which due to their characteristics may lead to interpretation inaccuracies. There is no doubt that health policy should be included in the set of policies referred to as public policies, which is suggested by minimalist definitions of health policy boiling down to the statement that it is a political process related to health [3]. If the above assumption is included in the concept of public policy proposed by Dye, then health policy should be interpreted as intentional actions (or omissions) of public authorities in the area of health [4]. The above interpretation method goes in a direction similar to the one expressed by the World Health Organization, which perceives health policy as decisions, plans and actions that are taken to achieve specific health protection objectives in society [5]. In this sense, it would require a separate interpretation if the abandonment of certain actions and ignoring some health problems should be perceived as another manifestation of deliberate action, or as a result of not recognizing health problems. There is no doubt however that in both cases the content of the actions will illustrate the focus of public authorities' attention.

Therefore, it is clearly seen that there is the possibility of looking at health policy from at least two separate perspectives. One would be the perspective of health problems and needs, while the other would be the perspective of activities of public authorities. This distinction, in the context of evaluation issues discussed later in the paper, seems to be of key importance. The first of the above perspectives, by including in its scope both areas of activity and omitted areas, will refer to the health condition of the population as the resultant of the current state. This gives the opportunity to consider health policy in the context of its complementarily, but at the same time, due to the huge range of problems that fall within the catalogue of health needs, it definitely makes it difficult to make reliable value judgments about it. On the other hand, focusing on areas of activity of public authorities leads to a clear facilitation of analytical operations, monitoring or evaluation, but at the same time limits the value of evaluation activities, causing them to describe only a fragment of reality. In this sense, health policy will take the form of programs and plans that in a holistic way (at least from the point of view of their authors) will aim to produce specific results. Health policy understood in this way does not have to constitute, and usually does not constitute, a monolithic structure, but splits into a number of policies/plans/programs, implemented by many decision makers at various levels. In the evaluation context, it should be emphasized that the subject of the assessment are then the partial programs, and not the overall picture of the state of health of the population. Analyzing literature dedicated to evaluation in health policy, one can observe a clear dominance of such an approach, which seems to coincide with the direction of development of this field also in relation to other sectors.

The way the evaluation is understood also needs to be clarified. Common understanding brings it down to giving grades or assigning values, however, this is a major simplification. There are many different views in the literature on the essence of evaluation. According to Scriven, this is a process that determines the value and benefits of objects, and the assessment of its product. Evaluation is not limited to accumulating and summarizing the data relevant to the decision to be taken, because it is only the first of its components. The second is to set appropriate standards and values based on which the assessment will be carried out [6]

In turn, according to Vendug, evaluation is considered to be a careful, retrospective assessment of the properties, values and advantages of administration, products and results of government intervention. In the approach proposed by the abovementioned the author evaluation is to play a role in shaping future practical solutions [7].

For the purposes of the practice, there are usually various forms of adaptation of the way the evaluation is expressed in order to emphasize its utilitarian character. An example would be United Kingdom (UK) Treasury Magenta Book Guidance for Evaluation, where the evaluation process was described as testing the implementation and impact of policies to assess whether the expected results, costs and benefits have actually been achieved [8]. Evaluation allows to determine what works, where problems arise, highlight good practices, indicate unintended effects or unforeseen results, and determine the effectiveness of expenses incurred. Similarly to Vendug’s approach, the translation of evaluation outcome into the course of future decision-making processes was also emphasized.

The distinguishing feature of evaluation in public policies is the fact that it is inherently inscribed in the classical model of the political process, constituting one of its separate stages [9, 10]. Usually, this stage is the last one, however, evaluation procedures also accompany earlier policy stages, or more precisely, each of these phases may be assessed not only after the closure of a single cycle, but also during its duration, depending on the adopted evaluation pattern. It is also worth emphasizing that evaluation itself is a political activity, which is strongly influenced by the values of individuals responsible for its design and conducting [11].

It is recognized that prior to specifying the set of properties, it is necessary to ensure the effectiveness of the evaluation. Firstly, the evaluation requires precise definition of its object and the time at which the evaluation should be carried out. Secondly, one should consider who will be conducting the evaluation and what competences they have for it. Thirdly, the criteria on the basis of which the evaluation will be carried out should be defined and an evaluation procedure should be developed.

In accordance with the theoretical considerations, evaluation is subject to numerous divisions, which illustrate the different approaches and the multidimensionality of the discussed process. According to E. Stern, methodological approaches to evaluation can be reduced to three main currents [12]:

- a position based on criteria or standards that deals with the assessment of success and results by applying standards;
- a causal inference standpoint that deals with explaining the impact of the program and success;
- a shaping or change-oriented position aimed at improving both programs and those who participate in them.

The purpose of this article is to discuss the main methodological approaches to evaluation and their potential application in health policy assessment. The evaluation methods discussed in the paper were identified on the basis of three criteria: time, purpose and scope.

## Material and methods

**Fig. 2:**
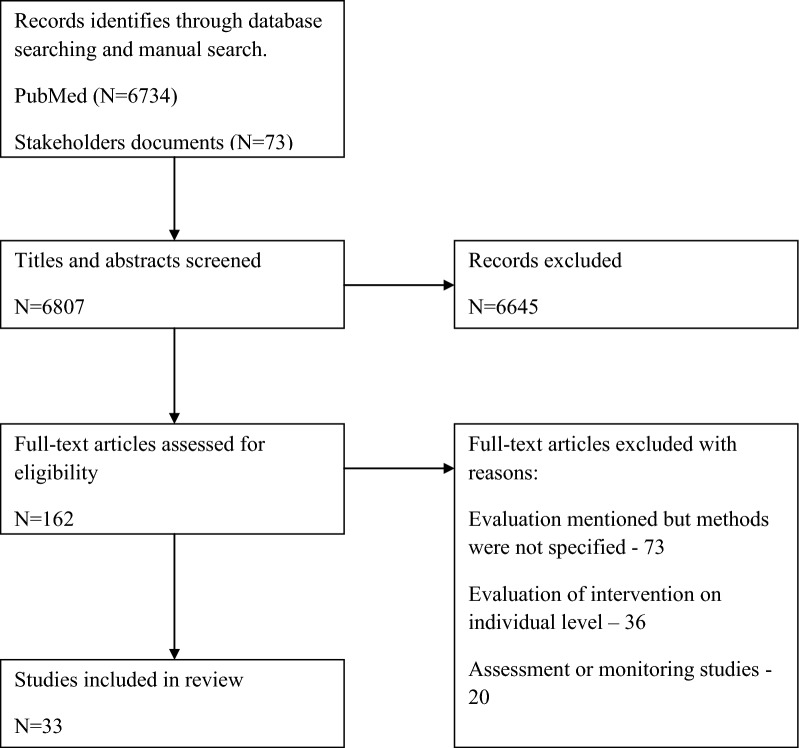
Review of flowchart

The study was based on non systematic, narrative literature review. Authors have conducted a literature search, using PubMed, Science Direct as well as manual search of reports, position papers, studies etc., produced by government, NGO’s, academia and other stakeholders, that were not published by commercial publishers (so called ‘grey literature’ [13]). To identify relevant items authors have conducted searches of websites of selected stakeholders involved in health policy evaluation activities (i.e. European Commission, World Bank, WHO and NGO’s). Both database searches and manual searches were limited to publications written in English. The following search terms were used: “evaluation types”; “evaluation method”; “assessment”; “health policy evaluation”. This search strategy led to 162 hits selected for eligibility check. After screening 129 were excluded due to insufficient description of evaluation methods, scale of evaluated intervention or focus on assessment and/or monitoring. As a result 33 items were qualified for further review. Selection process was depicted on a flowchart (Fig. 2). Overview of selected papers led to identification of following criteria for distinction of evaluation methods: time, purpose, scope of evaluation, evaluator, area. The table (Table 1) below presents a summary list of evaluation types with a brief description of the essence of each of these types [14–22]. Due to insufficient material on the methods distinguished on the basis of evaluator and area criteria authors have decided to exclude those methods from the scope of this study and focus on methods identified with the use of remaining three.

**Table 1:** Types of evaluation according to the main criteria of division

Criteria	Evaluation method	Characteristics
Time	Ex ante	Provides strategic information about the main choices at an early stage, when the possibility to influence the course of an undertaking is greatest Ex ante evaluation is a broad initial assessment aimed at identifying which alternative will yield the greatest benefit from an intended investment The goal of ex post evaluation is first and most importantly to assess the lessons learned in an undertaking
	Mid-term evaluation	It serves two immediate purposes: decision-making and taking stock of initial lessons from experience Provides a programme or project manager with a basis for identifying appropriate actions to: (a) address particular issues or problems in design, implementation and management, and (b) reinforce initiatives that demonstrate the potential for success
	Ex-post	Systematic and objective assessment of a completed project, programme or policy—in the context of their planning, implementation and obtained results Its objective is the determination of real effects and justification of intervention in a particular form
Purpose	Formative	Any evaluation activity directed at improving a programme's design, planning, development and implementation It is directed at ensuring independent constructively critical input into programme development
	Summative	Involves post-implementation assessment of the net and gross effects of the program
Scope	Meta-evaluation	An instrument used to aggregate findings from a series of evaluations Involves an evaluation of the quality of this series of evaluations and its adherence to established good practice in evaluation
	Cluster evaluation	Focuses on progress in achieving the overall objectives of the program initiative Covers a group of projects to identify common threads and topics that become more relevant after cross-confirmation
Evaluator	Independent	It provides an understanding of the following: How well a program has articulated its vision and is achieving its mission The quality of activities and how useful they are in meeting clients’ needs How capacities such as financial and executive management, governance and country-based resources affect quality
	Participatory	Researchers, facilitators, or professional evaluators collaborate in some way with individuals, groups, or communities who have a decided stake in the program, development project, or other entity being evaluated
Position in relation to the evaluated entity	External	Evaluation should be undertaken by non-staff members (‘external evaluators’)
	Internal	Evaluation should be undertaken by staff members (‘internal evaluators’)

Taking the above division as a framework for further considerations, it should be emphasized that it can be treated in an instrumental manner as a set of questions to be answered by the coordinators of all projects/programmes that could be subjected to evaluation judgment. The answers obtained in this way will be a premise for choosing the right path of evaluation. Due to the fact that the criteria adopted in the table above operate at various different levels, in fact they do not describe separate evaluation methods, but only describe the properties of evaluation processes that are actually carried out. At the stage of formulating assumptions for the evaluation of programs, policies or strategies, choices made in the scope of evaluation dimensions also to some extent determine the possibilities of choice in other dimensions.

## Results

### Barriers in the use of evaluation in the analysis of health policy as a sectoral policy

Attempts to apply evaluation methods to the overall assessment of health policy are subject to numerous limitations, which, if ignored, can lead to a complete undermining of the sense of making such efforts. Certainly, the problem that is most difficult to ignore is the fact that for health policy interpreted as a public policy sector it is difficult to indicate the objectives for which it is implemented. Referring to the previously quoted understanding of health policy as a public policy in Dye's interpretation, it can be seen that establishing its purpose would have to be arbitrary. Therefore, the evaluator would be forced to infer about the general purpose of health policy on the basis of the general direction of the intentions declared by public authorities, expressed in individual program documents, strategies, long-term plans etc. created in this way may prevent reproduction of the overall vision of health policy. Here, however, the question of intentional or unintentional selectivity in the actions of the state arises again, which means that the image of goals created in this way may make it impossible to recreate the overall vision of health policy. You can also try to evaluate based on a reference to externally set norms or standards (e.g. WHO guidelines or findings based on theoretical models), but in this case you should be aware that the actors directly involved in the design and implementation of health policy they do not have to treat these goals as values, and thus do not have to show their will to achieve them. Nevertheless, it seems that greater cognitive value is obtained by adopting the second of the proposed approaches, with notion that it does not have to (and often will not) entail practical consequences in the form of real impact on the shape of implemented policies.

Another caveat that should be made results from the fact that, while individual health policy programs have their own time frames, health policies taken as a whole are deprived of them. Thus, evaluation in such a context is always carried out during the course of health policy and can at best inform about the progress in achieving general objectives or, if we are able to indicate them, objectives adopted for a limited time frame (e.g. parliamentary term).

Having the above reservations in mind, we will focus on indicating the potential applications of various forms of evaluation, separated on the basis of previously defined criteria. Description of identified barriers and advantages of discussed methods is presented in table (Table 2).

**Table 2:** Potential and limitations of evaluation methods

Criteria	Evaluation method	Potential	Limitations
Time	Ex ante	Carried out at an early stage can have a positive impact on the overall policy implementation process	Lack of access to valid evidence from scientific research It is necessary to refer to other evidence whenever the policy objectives change
	Mid-term evaluation	Can lead to a number of changes in the program, project or policy, especially in the process of their implementation	There is a potential risk of making premature decisions about changes in the ongoing policy
	Ex-post	Provides evidence for decision making process	Difficulty to establish cause-and-effect relationships due to complexity of health policy The complexity of the institutional environment may impede the identification of valuable data sources for the evaluation process
Purpose	Formative	Allows for the necessary changes to be made to the programs to improve their efficiency	It’s value is largely dependent on the previously carried out summative evaluations for policies of a similar nature, scope and area of activity
	Summative	Linking activities with achievements It allows to infer about the effectiveness of implemented solutions	Does not provide grounds for drawing conclusions about what has failed in the implemented activities or what has proved helpful
Scope	Meta-evaluation	A basic set of evaluation criteria is easy to determine based on generally available standards of national or international evaluation societies Allows for elimination of evidence from substandard evaluations	Not identified
	Cluster evaluation	A scheme for evaluation is created to capture a broader picture of the issue	Solution created for project evaluation, i.e. easily identifiable projects that remain limited in time and have clearly defined objectives

### Time dimension

The temporal aspect of evaluation boils down to determining the right moment for conducting evaluation activities. As indicated in Table 1, basing on the above criterion, there are three main types of evaluation: ex-ante (prospective), mid-term and ex-post (retrospective). The application of the ex-ante evaluation method is currently practiced at the stage of designing activities. It seems that this type of evaluation can be considered quite commonly used in the area of health-related policies, although in the case of observed practice, it should be stipulated that this usually applies to health projects financed from public funds and selected in the grant application procedure [23]. This procedures usually include the stage of evaluation of applications according to predetermined criteria. In the case of programs or policies, usually such evaluation is replaced with impact assessment and expert consultations. These processes can hardly be considered as an equivalent of evaluation, mainly due to a different reference point (as in the case of impact assessment) or unstructured nature (as in the case of expert consultations, often not requiring them to apply a methodical approach). In international literature, the prospective evaluation procedure usually occurs in the context of investment decisions or research projects [24, 25]. The subject literature also lacks studies that would indicate the limitations of this type of evaluation while being used in health policy. Nevertheless, we could use arguments of a universal nature that come from studies carried out for the needs of other areas of public policy. The relatively frequent lack of access to valid evidence from scientific research should be considered the main factor limiting the usefulness of prospective methods. This phenomenon is particularly frequent and severe in relation to the assessment of the socio-economic context, but also the state of health and health behaviour of the population [26]. In addition, it should be noted that whenever the policy objectives change, be it in relation to its mechanics or in relation to the material and subjective scope, it is necessary to refer to other evidence.

Mid-term evaluation is mainly pragmatic, and could be brought down to ongoing verification of activities carried out and their value in relation to the objectives to which they were subordinated. It is worth mentioning that this is also a procedure that is relatively often confused with ongoing monitoring, therefore it should be noted that the latter is devoid of a axiological aspect, which is the essence of all evaluation activities [27]. Due to its formative nature, mid-term evaluation can lead to a number of changes in the program, project or policy, especially in the process of their implementation. The literature review did not identify any barriers that could be considered specific to this type of evaluation. However, it seems that in this case there is a potential risk of making premature decisions about the need to change the current mode of action.

Ex-post evaluation is a comprehensive summary of a program, project or policy, not only in relation to its results, but in all its aspects. The main challenges related to this type of evaluation include:

1. The increasing complexity of the analyzed phenomena, which may make it difficult to establish cause-and-effect relationships, and thus to make judgments about the value of actions taken under the program or policy in relation to the results obtained.
2. The complexity of the institutional environment, which may impede the identification of valuable data sources for the evaluation process.

Summing up the thread of the time criterion in relation to the evaluation process, it should be noted that the problem of lack of time frame is a heavy burden for the application of the forms of evaluation listed above. As it was mentioned in previous paragraphs, health policy when considered as a sectoral policy, does not have clearly defined time frames. Thus, keeping strictly to the nomenclature described above, each evaluation would be a mid-term evaluation. This does not exclude the possibility of using ex-ante and ex-post evaluations, however due to their very nature they can only relate to a selected time period. Therefore decision to make either one of those evaluations should be followed by indication of the relevant premises determining the setting of a time frame.

### Purpose dimension

Many authors who write about the topic of evaluation consider the purposiveness of evaluation activities as the decisive criterion, and therefore refer to the division of evaluation into formative and summative. Historically, it is also one of the oldest divisions proposed, introduced to literature in the 1960s by Scriven [6].

Formative evaluations are carried out during the development and implementation of the program, and their usefulness is manifested primarily in obtaining guidance on how to best achieve the objectives of the program or how to improve it. Summative evaluations are carried out when the programs are well established and allow determining to what extent the program achieves its goals.

Thus, formative evaluations are considered to be particularly useful during the implementation of pilot projects or the implementation of new strategies. It seems most advantageous to use them at early stages. The evaluation results obtained in this way allow the necessary changes to be made to the programs, in order to improve their efficiency [28]. The scope of such evaluation includes two areas of critical importance for the quality and value of planned interventions, which are needs assessment and process evaluation. It should be noted that such assumptions of formative evaluation are now an immanent part of many recommendations related to the planning and development of interventions in the field of health policy. Examples include A Planning Framework for Public Health Practice (National Public Health Partnership, Australia) [29] or The Health Promotion Strategic Framework (Health Service Executive, Ireland) [30]. In both cases it was pointed out that the development of a health intervention program should be initiated by recognising a health problem that is supposed to be a focal point of the activities carried out and identifying the determinants of the problem.

Summative evaluation focuses on the results of a project, program, strategy or policy. It also refers to the observed impact of the project on change in selected indicators (e.g. change in the incidence of specific disease entities). The logic of the program and the ability to see the difference between causation and coincidence become important in this case. The distinction between performance evaluation and impact assessment is highlighted, among others by CDC in its recommendations for stakeholders involved in the implementation of the Healthy Communities program. In the discussed proposal, this division was referred to the time dimension and it leads to recognizing the impact evaluation as an assessment of long-term effects, while the outcome evaluation was referred to short- and medium-term effects only [31].

At this point, one should pay attention to the rather special situation that occurs in the case of the second of the forms discussed. If negative deviation from the expected outcome is observed it must necessarily lead to the initiation of formative activities. Otherwise, an irrational situation would arise in which the observed ineffectiveness or even harmfulness of implemented policies, programs or interventions would not entail any corrective actions or conclusions for the future [17, 32].

Regardless of any kind of digressions, summative evaluation presents a special value for decision-makers, because, by linking activities with achievements, it allows to infer about the effectiveness of implemented solutions. Thus, its formative properties will also be manifested in a broader aspect, resulting from the fact that it provides the basis (evidence of effectiveness) for continuing specific actions and programs or for their termination. Auer and Kruppe among the others pay attention to the feedback between the results of the evaluation and its impact on the shape of the agenda of future activities (in their cases related to the labour market policy) [33]. In the conditions of the functioning of democratic mechanisms and the pursuit of transparency in spending public funds, any relevant decision needs to be based on solid substantive foundations. This seems particularly important in the case of spending funds on health programs, the results of which are often not directly experienced by the public, which in turn leads to raising questions about rationality of incurring such expenses.

In current practice, the tendency to introduce summative methods can be clearly seen at the level of national or international health strategies. As a primary example one can indicate, among others, evaluation activities conducted by WHO as part of monitoring and evaluation of the Health for All strategy or similar activities related to the implementation of the Sustainable Development Goals. In both cases, the evaluation activities are based on reference to the goals and measures adopted at the stage of building the strategy, describing current state of their implementation. At the same time, there is an overlap between two dividing lines, because the evaluation is carried out at both global and national levels. The choice of evaluation approach was influenced by the high complexity of the analyzed matter, as well as the diversity of situations in individual countries and regions [34, 35]. In this case, summative evaluation, as focused mainly on results, allows to omit the context of the conducted activities.

Regarding the barriers of formative and summative evaluation, it should be noted that in this case the strong relationship between both forms is emphasized. The value of formative evaluation is largely dependent on the previously carried out summative evaluations for programs of a similar nature, scope and area of activity. At the same time, it should be emphasized that, unlike the formative evaluation, the summative approach does not provide grounds for drawing conclusions about what has failed in the implemented activities or what has proved helpful, due to the fact that it focuses on assessing the outcomes of the program in relation to previously defined goals.

The use of the formative or summative nature of evaluation for a holistic assessment of health policy raises several questions. In a first place we need to ask what will be the benchmark for formative evaluation? If we assume that formative evaluation should be supported by evidence from summative evaluations, then the question should be asked which summative evaluations will meet the conditions allowing to consider them as valid evidence? If we take a look at the level of health programs, then there is observable common practice of utilizing reference points in the form of experiences of other countries (or regions, or international programs) introducing similar solutions in environments with similar specificity. While such operations, even on a project or program scale, are subject to significant risk, there is doubt whether they can be carried out at all if we aim at adopting holistic approach to health policy. This is due both to the incomparability of the conditions in which the policy is implemented and to the complexity of the policy itself. In this sense, the scope constituting the subject of evaluation would have to be limited at best to selected components of health policy. A similarly troublesome issue may be the issue of reference to the goal, which, as indicated earlier, in the case of overall health policy is not clearly defined, and if attempts to determine it are made, the level of generality of the proposed interpretations would hamper the implementation of evaluation studies.

### The scope of the evaluation

Within the scope of the evaluation criterion, meta-evaluation and cluster evaluation deserve to be singled out. The first of these forms is used to determine the value of evaluation in relation to ethical, methodological and praxeological standards. Any evaluation can be subject to this type of assessment, moreover, a basic set of evaluation criteria is easy to determine based on generally available standards of national or international evaluation societies (e.g. PTE standards) [36]. The use of meta-evaluation brings with it the advantages of particular utility, primarily in the context of designing activities in the field of health policy, allowing for explicit rejection as potential premises of these evaluation studies that do not meet the abovementioned standards.

The issue of cluster evaluation is slightly different and is assumed to be a solution to the problem of the complexity of the evaluated issues. The method created by the Kellog Foundation is mainly used to evaluate projects that are subject to one problem area and are implemented in parallel. The cluster then includes a set of projects, each of which is subject to evaluation, however, the very assumptions of the evaluation are formulated at the cluster level, not individual projects level. Due to the unification of assumptions and the possibility of adapting them to the evaluation needs of specific projects, a scheme is created to capture a broader picture of the issue [18]. This would not be the case if the evaluation was transferred into a series of unrelated operations dedicated to individual projects. Undoubtedly, the limitation of the aforementioned form of evaluation is the fact that it was created as a solution for project evaluation, i.e. easily identifiable projects that remain limited in time and have clearly defined objectives. However, it seems that the philosophy of cluster evaluation itself presents the potential that can be used in the overall assessment of health policy. Identifying the components of the health policy "cluster" would remain a major challenge.

## Discussion

The issue of comprehensive health policy assessment has not been discussed in the literature so far. However, this problem is part of similar discussions in the area of public health. In this regard, it is worth referring to the publication of Rutter et al., which argues that the identification, implementation and evaluation of effective solutions to major public health threats requires a broader, comprehensive approach. These authors indicate that current practice is mainly limited by the dominance of a simplified, linear model of inference, which makes it difficult to take into account the way in which processes and their results contribute to systemic changes at every stage. At the same time, they suggest instead of asking whether intervention works to solve the problem, policymakers should rather determine if and how it contributes to transforming the system in a beneficial way [37]. Smith and Petticrew write even more directly about the need to change the current approach, noting that current practice has been based rather on microanalysis focused on the individual and health sector, largely ignoring the macroanalysis of social and systemic conditions. The same authors have identified three main challenges to the widespread use of the new model, which are: (1) determining and evaluating results; (2) identifying and understanding complex causal pathways in social interventions, and (3) developing a multi-sectoral assessment to meet the information needs of stakeholders [38].

In the context of the discussed problem, it is also worth noting that any division of evaluation methods overlaps with the issue of using two different types of evidence in evaluation activities—quantitative and qualitative. If we look at the evaluation processes from this perspective, then it can be noticed that in practice used by decision-makers in health policy, as in most public policies, there seems to be a tendency towards basing decisions on the first of these categories [39, 40]. It should be emphasized, however, that the above observation is only a hypothesis, the verification of which goes beyond the scope of this publication and would require more extensive research. Nevertheless, the fact of using a diverse set of evidence for the purposes of evaluation processes forces evaluators to use tools and techniques derived from many scientific disciplines. Bearing in mind that the implementation of health policy is largely based on financing from public funds, particular attention should be paid to the impact of health economics in this respect. By developing methods of economic evaluation (including cost-effectiveness assessment and cost–benefit analysis), health economics provides both the necessary evidence and tools for interpreting the collected information [41–43]. It seems that in democratic societies whose economic model is based on a free market economy, this category of evidence may be one of the leading factors in the decision-making process.

The need for a new approach in public health is also aptly described by South et al. Noting that it stems somewhat from the growing frustration caused by the limitations of traditional models, as well as from a better understanding that "that real world public health (…) needs multi-sectoral action to address the causes of the causes of poor health" [38].

To a large extent, the allegations are related to the long-term primacy of randomized controlled trials (RCTs), which were considered to be the most reliable and expected methods for verifying the effectiveness of public health interventions. Without undermining their cognitive value, it can be pointed out after Moor et al. that many researchers question the usefulness of this method for assessing interventions implemented in complex social systems [44]. At the same time, these reservations seem to affect recommended practice and standards, as evidenced by changes introduced in the recommendations of the Medical Research Council in 2008 and 2012 [44, 45]. In the context of the issues discussed in this paper, it is worth noting that the above-mentioned recommendations emphasized the value of process evaluation as no less important than assessment of results. The wider the intervention, the more difficult it is to evaluate it, which results from the limited utility of standard evaluation methods discussed earlier (for RCTs). Basu et al. drew attention to other aspects of complications related to the evaluation of interventions and policies undertaken on a larger scale, indicating three main issues: (1) distinguishing the effect of the policy from time trends in health outcomes or existing differences between politically-affected and non-affected communities ( using difference approaches); (2) constructing a comparative population when the policy affects a population for which a well-chosen comparator is not immediately available (using propensity assessment or synthetic control methods); (3) responding to imperceptible interfering factors by applying quasi-random changes in policy exposure (using regression discontinuities, instrumental variables or short-range matching approaches) [46].

Evaluation processes in health policy will gradually gain importance in coming years, which will be fostered by both the growing demand for health services, stimulated by demographic changes, the increased incidence of chronic diseases that are costly to treat, and the restrains of public funds allocated to health [47]. At the same time Baum et al. point out to the fact that knowledge of how to evaluate health policy is not sufficiently widespread so far, and there is a particularly lack of studies that would address this issue from the point of view of middle and low income countries [48]. Nevertheless there is an increasing number of studies proving that the resources allocated to healthcare and public health in this group of countries are not sufficient to meet the growing demand [49–51]. Recent analyzes carried out in the group of highly developed countries indicate that also in their case there is a serious threat to the maintenance of the efficiency of health systems based on public funding. In the context of European countries, this problem was raised, among others by Jakovljevic et al. who indicated the need to improve the efficiency of financing health care [52].

At the same time, evaluation must be based on evidence, which is often missing not only in developing countries, but also in highly developed countries. For example, this problem in the context of financing research in the United Kingdom was indicated, inter alia, by Hunter [53] and Rutter et al [37]. However it is not only the evidence that is missing, but there is also a problem of insufficient development of instruments used in the evaluation process. If evaluation is to present value for the decision-making process, then it seems necessary that it includes all the most important aspects of the evaluated policy. However, while in the case of the financial aspect there is a well-developed and well-established set of evaluation tools developed in the field of health economics [54], in the case of assessing the social impact of implemented policies, there are very limited tools available.

With reference to the subject of this publication, it should be noted that a significant limitation to the discussion currently conducted in the area of public health is the fact that the gathered evidence comes from the evaluation of intervention, which in itself does not have to be characterized by a high degree of complexity. Quite frequently those are scaled at the level of community, or even narrowed down to the individual or household level (although this is not the rule and may take the form of wider population programs). In this respect, the complication to be considered, when attempting to evaluate health policy as a whole, is not only the fact that it functions in a complex system but also the fact that its components cannot be easily identified. This is the result of the wide scope of the subject of health policy and the complexity of its links with other public policies.

The problem of the scale of conducted activities was also addressed in its recommendations by the CDC, pointing to the differences between policy evaluation and program evaluation. These recommendations recognize that evaluation at the policy level requires a systemic approach, and the degree of control by decision makers is lower than in the case of health programs [55].

## Conclusions

There is no doubt that each of the approaches to evaluation, regardless of the criterion on the basis of which they were identified, presents a useful value in relation to the evaluation of health policy. However, decision making process should be based on the awareness of the limitations of each approach. Given the complexity of the matter of the health policy, attempts aimed at its overall assessment should be based on an approach integrating various methods, while ensuring coordination of evaluation activities and making them subordinated to common assumptions. The current practice of simplifying the complexity through the evaluation of health policy components, seems to be the most rational solution. However, without placing such perceived evaluation efforts in a broader context there is a risk that instead of a comprehensive picture of the state of health policy we will receive a set of unrelated elements that give a distorted reflection of reality. Hence, it seems that the issue of the scope of activities should be of particular interest to both decision-makers and other stakeholders.

## Data Availability

Not applicable.

## Abbreviations

CDC: Centers for Disease Control and Prevention
NGO: Nongovernment organisation
PTE: Polish Evaluation Society (in Polish: Polskie Towarzystwo Ewaluacyjne)
RCT: Randomized controlled trail
WHO: World Health Organization

## References

[1] Easton D (1953). The political system: an inquiry into the state of political science.

[2] Reichardt I. Ewaluacja jako narzędzie analizy polityk publicznych. Zarządzanie Publiczne; Numer 1. 2011;1(13):11–3. https://www.ejournals.eu/Zarzadzanie-Publiczne/2011/Zarzadzanie-Publiczne-1-2011/art/1881/.

[3] Włodarczyk CW (2014). Współczesna polityka zdrowotna: wybrane zagadnienia.

[4] Dye TR. Understanding public policy. Fifth edition. Englewood Cliffs: Prentice-Hall, [1984] ©1984; https://search.library.wisc.edu/catalog/999531243702121

[5] Health Policy. https://www.who.int/topics/health_policy/en/. Accessed Mar 23 2020

[6] Scriven M (1991). Evaluation thesaurus.

[7] Vedung E. Public policy and program evaluation. Administrative Science Quarterly. 1997;44.

[8] United Kingdom (UK) Treasury. United Kingdom (UK) Treasury Magenta Book Guidance for Evaluation. 2011. https://assets.publishing.service.gov.uk/government/uploads/system/uploads/attachment_data/file/879438/HMT_Magenta_Book.pdf. Accessed 3 Apr 2020.

[9] Warner J, Wegrich K, Fischer, Frank, Mille GJ (2007). Theories of the policy cycle. Handbook of public policy analysis theory, politics and methods.

[10] Ronit K, Porter T. Harold D. Lasswell. The decision process: seven categories of functional analysis. In: Lodge M, Page EC, Balla SJ, editors. The Oxford Handbook of Classics in Public Policy and Administration. 2015.

[11] Patton MQ (1988). Six honest serving men for evaluation. Stud Educ Eval..

[12] Stern E. Philosophies and types of evaluation research. The Foundations of Evaluation and Impact Research. Third report on vocational training research in Europe: Background report. 2004. https://www.cedefop.europa.eu/files/BgR1_Stern.pdf.

[13] Adams J, Hillier-Brown FC, Moore HJ, Lake AA, Araujo-Soares V, White M (2016). Searching and synthesising “grey literature” and “grey information” in public health: critical reflections on three case studies. Syst Rev..

[14] Samset K, Christensen T (2017). Ex Ante Project evaluation and the complexity of early decision-making. Public Organ Rev..

[15] Duignan P. The use of formative evaluation by government agencies strategic evaluation working paper first posted on 3 June 2004 as Version 1-2-4. 2004. https://www.strategicevaluation.info/se/documents/121pdff.html. Accessed Feb 22 2020

[16] Janus M, Brinkman S. Evaluating early childhood education and care programs. In: Peterson P, Baker E, McGaw BBT-IE of E (Third E, editors). Oxford: Elsevier; 2010. p. 25–31.

[17] Frey BB. The SAGE encyclopedia of educational research, measurement, and evaluation. Thousand Oaks, California; 2018. https://methods.sagepub.com/reference/the-sage-encyclopedia-of-educational-research-measurement-and-evaluation

[18] Mathison S. Encyclopedia of Evaluation. Thousand Oaks, California; 2005. https://methods.sagepub.com/reference/encyclopedia-of-evaluation

[19] World Bank. World bank group evaluation principles. Washington, DC; 2019. https://ieg.worldbankgroup.org/sites/default/files/Data/reports/WorldBankEvaluationPrinciples.pdf

[20] Cousins JB, Whitmore E (1998). Framing participatory evaluation. New Dir Eval..

[21] Calidoni-Lundberg F. Working Paper R2006:002 Evaluation: definitions, methods and models. An ITPS framework. 2006.

[22] Capeling-Alakija S, Benbouali A, Brewka B, Diallo D. Results-oriented monitoring and evaluation a handbook for programme managers. 1997. https://web.undp.org/evaluation/documents/mec25.htm

[23] Gallo S, Thompson L, Schmaling K, Glisson S (2018). Risk evaluation in peer review of grant applications. Environ Syst Decis..

[24] Barretta A, Ruggiero P (2008). Ex-ante evaluation of PFIs within the Italian health-care sector: What is the basis for this PPP?. Health Policy..

[25] Meacock R (2018). Methods for the economic evaluation of changes to the organisation and delivery of health services: principal challenges and recommendations. Health Econ Policy Law.

[26] Banks R. Ex-Ante-Evaluations: Strengths, weaknesses and opportunities. Paper prepared for the European Commission’s Edinburgh Conference, Evaluation for Quality, 4th European Conference on Evaluation of the Structural Funds, 18–19 September 2000. Edinburgh; 2000. https://ec.europa.eu/regional_policy/archive/sources/docconf/edimbourg/pdf/banks_en.pdf.

[27] OECD/DAC. Glossary of Key Terms in Evaluating and Results-based Management. 2002. https://www.oecd.org/development/peer-reviews/2754804.pdf. Accessed Mar 2 2020.

[28] UNFPA Evaluation Office. Formative evaluation of the UNFPA innovation initiative. New York, NY; 2016. https://www.unfpa.org/sites/default/files/admin-resource/Inception_Report_UNFPA_Formative_evaluation_innovation_initiative_v2.pdf.

[29] Partnership National Public Health. A Planning Framework for Public Health Practice. Melbourne, Australia; 2000. https://www.health.nsw.gov.au/research/Documents/planning-framework.pdf.

[30] Health Service Executive Ireland. The Health Promotion Strategic Framework. 2011. https://www.healthpromotion.ie/health/inner/health_promotion_strategic_framework. Accessed Feb 26 2020.

[31] CDC. Building Our Understanding: Key Concepts of Evaluation. What is it and how do you do it? https://www.cdc.gov/nccdphp/dch/programs/healthycommunitiesprogram/tools/pdf/eval_planning.pdf. Accessed Feb 25 2020.

[32] Sambo LG, Kirigia JM (2001). Evaluation of health-related programmes in Africa: a vision for 2020. East Afr Med J.

[33] Auer P, Kruppe T. Monitoring of labour market policies in EU member states. In: Lange T, editor. 1st ed. Cheltenham, UK ; Northampton, Mass.: Edward Elgar Pub; 1996. p. 164–77.

[34] Bryant JH (1988). Health for all: the dream and the reality. World Health Forum.

[35] Asma S, Lozano R, Chatterji S, Swaminathan S, de Fátima MM, Yamamoto N (2020). Monitoring the health-related Sustainable Development Goals: lessons learned and recommendations for improved measurement. Lancet.

[36] Polish Evaluation Society. Polish evaluation society—evaluation standards. Warsaw; 2008. p. 1–8. https://pte.org.pl/wp-content/uploads/2015/08/PTE_Evaluation_Standards.pdf.

[37] Rutter H, Savona N, Glonti K, Bibby J, Cummins S, Finegood DT (2017). The need for a complex systems model of evidence for public health. Lancet..

[38] Smith RD, Petticrew M (2010). Public health evaluation in the twenty-first century: time to see the wood as well as the trees. J Public Health..

[39] Newman K, Fisher C, Shaxson L (2012). Stimulating demand for research evidence: What role for capacity-building?. IDS Bull..

[40] Hawkes S, Aulakh KB, Jadeja N, Jimenez M, Buse K, Anwar I (2016). Strengthening capacity to apply health research evidence in policy making: experience from four countries. Health Policy Plan..

[41] Oladimeji B, Muhamad A, Juni MH, Bolarinwa O, Juni M, Muhamad H (2015). Health economics evaluation in health planning. Int J Public Health Clin Sci.

[42] Goeree R, Diaby V (2013). Introduction to health economics and decision-making: Is economics relevant for the frontline clinician?. Best Pract Res Clin Gastroenterol..

[43] Dang A, Likhar N, Alok U (2016). Importance of economic evaluation in health care: an Indian perspective. Value Heal Reg Issues..

[44] Moore GF, Evans RE, Hawkins J, Littlecott H, Melendez-Torres GJ, Bonell C (2018). From complex social interventions to interventions in complex social systems: future directions and unresolved questions for intervention development and evaluation. Evaluation..

[45] Craig P, Dieppe P, Macintyre S, Michie S, Nazareth I, Petticrew M (2008). Developing and evaluating complex interventions: the new Medical Research Council guidance. BMJ..

[46] Basu S, Meghani A, Siddiqi A (2017). Evaluating the health impact of large-scale public policy changes: classical and novel approaches. Annu Rev Public Health..

[47] Jakovljevic M, Jakab M, Gerdtham U, McDaid D, Ogura S, Varavikova E (2019). Comparative financing analysis and political economy of noncommunicable diseases. J Med Econ..

[48] Baum F, Lawless A, Delany T, Macdougall C, Williams C, Broderick D (2014). Evaluation of health in all policies: concept, theory and application. Health Promot Int..

[49] Arun J, Kumar D (2016). Public health expenditure of BRICS countries—an empirical analysis. Int J Med Sci Public Health.

[50] Jakovljevic MM (2016). Comparison of historical medical spending patterns among the BRICS and G7. J Med Econ.

[51] Ranic N, Jakovljevic MM. Long term health spending alongside population aging in N-11 emerging nations. East Eur Bus Econ J. 2016;2(1):2–26. https://ideas.repec.org/a/eeb/articl/v2y2016n1p2-26.html.

[52] Jakovljevic M, Fernandes PO, Teixeira JP, Rancic N, Timofeyev Y, Reshetnikov V (2019). Underlying differences in health spending within the World Health Organization Europe Region-Comparing EU15, EU Post-2004, CIS, EU Candidate, and CARINFONET Countries. Int J Environ Res Public Health..

[53] Hunter DJ, Peckham S, Gadsby EW. Importance of Process and Impact Evaluation of Public Health Programmes/Policies Overall and Especially in Financially Deprived Settings Working Paper 1. Canterbury; 2017. Report No.: 1. https://www.kent.ac.uk/chss/docs/PublicHealthUkraine/Ch_2_Importance_Process_Impact_Eval_PHProgrammes.pdf.

[54] Jones AM, Rice N. Econometric Evaluation of Health Policies. In: Glied S, Smith PC, editors. The Oxford Handbook of Health Economics. Oxford University Press; 2011. p. 889–923. https://oxfordhandbooks.com/view/10.1093/oxfordhb/9780199238828.001.0001/oxfordhb-9780199238828-e-37. Accessed Sept 28 2020.

[55] CDC. Step by step—evaluating violence and injury prevention policies. https://www.cdc.gov/injury/pdfs/policy/Brief1-a.pdf. Accessed 4 Sept 2020.

